# Providing Care From Afar: A Growing Yet Understudied Phenomenon in the Caregiving Field

**DOI:** 10.3389/fpsyg.2020.00681

**Published:** 2020-04-15

**Authors:** Eva Bei, Orit Rotem-Mindali, Noa Vilchinsky

**Affiliations:** ^1^Department of Psychology, Bar-Ilan University, Ramat Gan, Israel; ^2^Department of Geography and Environment, Bar-Ilan University, Ramat Gan, Israel

**Keywords:** geographic distance, distance caregivers, distance caregiving, caregivers, chronic illness

Given the aging population and increasing life expectancy, the need on the part of older and ill populations for long-term care has risen rapidly (Roth et al., 2015). As a result, unpaid informal care is becoming even more important for health and social care delivery worldwide. Informal care refers to the provision of unpaid care to a relative or friend with a chronic illness, disability, or other long-lasting health needs (Revenson et al., 2016). A substantial body of literature has documented the psychosocial and physical consequences of the caregiving role. Recent studies have reported caregiver burden and strain as a multidimensional response to the psychological, physical, and financial stressors associated with the caregiving experience (Chiao et al., 2015; Faronbi et al., 2019). Emotional distress, anxiety, and impaired self-care are also commonplace among caregivers, attributed to the caregiving demands engendered by the care recipient's illness (Schulz et al., 2008; Bauer and Sousa-Poza, 2015).

The increased mobilization of our society and globalization of the workplace have resulted in additional challenges for many of these informal caregivers, especially adult children of aging parents; that is, difficulties that stem from living far away from the care recipients (Baldock, 2000; Bevan and Sparks, 2011). It is estimated that ~15–20% of all informal caregivers are distance caregivers (DCGs) (Douglas et al., 2016). The reasons for the geographic distance might include career or education, military deployment, divorce, or a simple life choice (Stafford, 2004).

DCGs engage in many supportive activities to meet the needs of their loved ones, such as assisting with financial and bureaucratic issues, providing social and emotional support, and even performing practical and nursing tasks, which, naturally, are more difficult to perform from a geographic distance (Parker et al., 2006; Cagle and Munn, 2012). In fact, nearly three quarters of DCGs assist with instrumental activities of daily living (IADLs), such as managing medications, arranging transportation, doing housework, and coordinating care with the local support of family, friends, or paid carers (Koerin and Harrigan, 2015).

Despite the above, current research on the unique needs and experiences of DCGs is limited. In the few studies conducted, DCGs have reported feelings of anxiety, stress, helplessness, and guilt related to their geographic distance from the care recipient (Schoonover et al., 1988; Mazanec, 2009). Experiencing the added stressors associated with caring from a distance, DCGs have also reported higher levels of uncertainty, inadequacy, and distress, especially if their resources for travel are limited (Harrigan and Koerin, 2007; Douglas et al., 2016). The National Alliance for Caregiving and American Association of Retired Persons survey (National Alliance for Caregiving/American Association of Retired Persons, 2004) revealed a critical association between caring from a geographic distance and stress, with 47% of caregivers who lived farthest away reporting emotional stress, compared to 43% of those who lived with the care-recipient on the same premises, and 28% of those who lived close to the care-recipient but not with him/her.

Thus, the aim of the current opinion paper is to shed light on this substantial population of carers. We claim that much more theoretical, empirical, and practical attention should be directed toward this caregiving context, in order to identify the unique needs and experiences of these caregivers and provide evidence for tailored interventions. First and foremost, the lack of a clear definition of the term DCG is a major lacuna in the field and should be addressed. In the few studies conducted, researchers have used mileage or travel time in order to define distance caregiving (Mazanec, 2012). However, it might not be appropriate to define DCGs according to travel time, as the amount of time it takes to get to a care recipient's home is dependent on a number of other factors including access to transportation, geographic barriers (i.e., difficult-to-navigate roads), and travel costs. In addition, evidence has shown that even among those who are identified as DCGs, the caregiving experience and outcomes might vary based on the exact geographic distance from the care recipient, with those living very far away reporting higher levels of emotional distress compared to those who live relatively closer to their care recipient (National Alliance for Caregiving/American Association of Retired Persons, 2004). For instance, previous studies suggested that those living more than 1 h away from the care recipient should be defined as long-distance caregivers (LDCGs) and not simply as DCGs (Cagle and Munn, 2012).

We therefore suggest the use of a common definition that would differentiate local carers from DCGs, and DCGs from LDCGs, based not only on the geographic distance and travel time, but also the mode of travel, caregiver's economic status, travel costs, mobility difficulties, perception of distance, and other barriers that might limit DCGs' and LDCGs' access to the care recipient. In the future, this differentiation could be achieved by the construction of a self-report questionnaire consisting of caregivers' socioeconomic and sociocultural factors as well as geographic barriers, mobility difficulties, and perception of distance. Mapping these dimensions will enable the identification of different caregiving profiles. The common use of such an instrument in caregiving research would allow for the differentiation between local caregivers and DCGs, but also between DCGs and LDCGs, with the aim of defining, in a consensual manner, the eligibility criteria of what constitutes a local caregiver, DCG, or LDCG.

Second, more research focusing on the unique psychosocial and health outcomes of DCGs/LDCGs is needed. Most of the studies conducted so far have been limited to cross-sectional data or secondary analysis of large data sets (Cagle and Munn, 2012; Douglas et al., 2016). More methodologically rigorous study designs such as longitudinal, diary, and mixed-methods studies should be employed in order to understand the specific advantages and burdens associated with distance caregiving, over the course of the care recipient's illness. These studies would likely demonstrate the impact of geographic proximity on DCGs/LDCGs' emotional distress, strain, quality of life, and other outcomes, over time. Also, as most of the research has involved educated, white, English-speaking participants, future studies should include more diverse populations, taking into account crucial cultural and sociodemographic factors including ethnicity/race, education, employment, and socioeconomic status, as well as cultural values and worldviews that might shape the phenomenon of distance caregiving (Cagle and Munn, 2012).

Finally, geographically sensible and tailored interventions, based on randomized controlled trials, are needed to support the unique needs of DCGs/LDCGs and their care recipients. Although a number of psychosocial interventions have been developed to support local caregivers and reduce caregiver burden, little empirical work exists that investigates feasible interventions for those caring from afar and their loved ones (Benefield and Beck, 2007; Blackstone et al., 2019). Technology-based and eHealth interventions may be especially useful in this respect, as they render the distance barrier irrelevant (Benefield and Beck, 2007; Fairchild et al., 2019). Yet, we suggest that future eHealth interventions should adopt a *dyadic coping model*, in which distance caregiving is perceived as a dynamic process, involving both DCGs/LDCGs and their care recipients (Bodenmann et al., 2019). Dyadic coping research claims that chronic illness poses a major stressor for *both* patients and their caregivers and that both members of the dyad are involved in a mutual coping process (Revenson et al., 2016). Therefore, online dyadic interventions should be adapted to the DCG population to improve communication between the two members of the caregiving dyad, focusing on the needs and stressors of both members and using problem-solving abilities for issues that arise from the distance caregiving situation (Revenson et al., 2016). In addition, Information and Communication Technologies (ICT), such as videoconferencing and monitoring technologies, should be developed to alleviate distress and reduce practical burdens by allowing DCGs/LDCGs more active participation in their loved one's care, regardless of geographic distance (Blackstone et al., 2019).

To conclude, DCGs are a growing, yet understudied subpopulation of caregivers. Unlike local caregiving research, studies on distance caregiving and long-distance caregiving are scarce, with inconsistencies regarding what even constitutes “distance.” More empirical research is needed to shed light on the unique needs and experiences of this population, using a consistent definition to facilitate comparison across studies and provide evidence for tailored interventions. Finally, technologically advanced and online dyadic interventions should be adapted to the unique needs of DCGs/LDCGs in order to bridge the distance between them and their loved ones.

## Author Contributions

All authors listed have made a substantial, direct and intellectual contribution to the work, and approved it for publication.

### Conflict of Interest

The authors declare that the research was conducted in the absence of any commercial or financial relationships that could be construed as a potential conflict of interest.
